# Efficacy and survival outcome of allogeneic stem-cell transplantation in multiple myeloma: meta-analysis in the recent 10 years

**DOI:** 10.3389/fonc.2024.1341631

**Published:** 2024-07-31

**Authors:** Si Yu Lin, Ke Jie Lu, Xiao Na Zheng, Jian Hou, Ting Ting Liu

**Affiliations:** Department of Hematology, Renji Hospital, School of Medicine, Shanghai Jiaotong University, Shanghai, China

**Keywords:** multiple myeloma, allogeneic stem cell transplantation, response rate, survival outcome, OS, PFS

## Abstract

**Introduction:**

Allogeneic hematopoietic cell transplantation (alloHCT) possessed direct cytotoxicity and graft-versus-multiple myeloma effect (GvMM). Growing trials have shown survival benefits of performing alloHCT in both newly diagnosed and relapsed MM.

**Methods:**

We aimed to provide a comprehensive analysis in the recent 10 years to verify the efficacy and survival outcome of alloHCT in MM patients. A total of 61 studies which provide data between 14/04/2013 and 14/04/2023 and a total of 15,294 data from MM patients who had undergone alloSCT were included in our study. The best response rates (CR, VGPR, PR) and survival outcomes (1-, 2-, 3-,5-, and 10-year OS, PFS, NRM) were assessed. We further conducted meta-analysis in the NDMM/frontline setting and RRMM/salvage setting independently.

**Results:**

The pooled estimate CR, VGPR, and PR rates were 0.45, 0.21, and 0.24, respectively. The pooled estimates of 1-, 2-, 3-, 5-, and 10-year OS were 0.69, 0.57, 0.45, 0.45, and 0.36, respectively; the pooled estimates of 1-, 2-, 3-, 5-, and 10-year PFS were 0.47, 0.35, 0.24, 0.25, and 0.28, respectively; and the pooled estimates of 1-, 2-, 3-, 5-, and 10-year NRM were 0.16, 0.21, 0.16, 0.20, and 0.15, respectively. In the NDMM/upfront setting, the pooled estimate CR rate was 0.54, and those for 5-year OS, PFS, and NRM were 0.69, 0.40, and 0.11, respectively. In a relapsed setting, the pooled estimate CR rate was 0.31, and those for 5-year OS, PFS, and NRM were 0.24, 0.10, and 0.15, respectively.

**Discussion:**

Our results showed constant OS, PFS, and NRM from the third year onwards till the 10th year, suggesting that alloSCT has sustained survival benefits. Good response rate and promising survival outcome were observed in the NDMM/ frontline setting.

**Conclusion:**

Although comparing with other treatments, alloSCT had a lower response rate and poorer short-term survival outcome, long-term follow-up could reveal survival benefits of alloSCT in MM patients.

## Introduction

Multiple myeloma (MM), the second most common hematological malignancy, is a monoclonal tumor characterized by the expansion of malignant plasma cells in the bone marrow (BM) (1). Uncontrolled expansion interferes with osteogenesis in BM, leading to lytic bone disease (2). Moreover, progression of the disease may lead to acute kidney injury, anemia, and hypercalcemia (3).

Drug resistance arises due to the intratumor high heterogeneity nature of MM (2). Recently, the standard treatment for transplant eligible MM patients is a combination of induction therapy (injectable proteasome inhibitor, oral immunomodulatory agent, and dexamethasone) and autologous hematopoietic stem cell transplantation (autoSCT) followed by lenalidomide maintenance therapy (3).

Allogeneic hematopoietic cell transplantation (alloHCT) possesses direct cytotoxicity and graft-versus-multiple myeloma effect (GvMM) (4). It remains controversial due to the high occurrence of graft-versus-host disease (GVHD), treatment-related mortality (TRM), and relapse rate. Recent data showed the crucial role of GVHD in the GVMM effect, specifically chronic GVHD (5). In research comparing survival between auto-auto and auto-allo after induction therapy, results showed higher overall survival (OS) and progression-free survival (PFS) in the auto-allo group. Moreover, there was a higher non-relapsed mortality (NRM) and lower risk of disease progression in the auto-allo group, verifying the continuous GVMM effect on disease control (6).

In a phase 3 trial comparing auto-auto versus auto-alloSCT in newly diagnosed MM (NDMM) patients with high-risk cytogenetic abnormalities (del13q, del17p), patients undergoing auto-alloSCT had a much higher median PFS and OS, showing the effectiveness of prolonged GVMM effect in improving survival in high-risk NDMM patients (7). The BMT CTN 0102 trial with long-term follow-up over 10 years showed a reduction in relapse, better PFS, and similar OS among high-risk NDMM patients treated with auto-allo. This trial further revealed the potential of alloSCT in overcoming the deleterious effect brought by high-risk cytogenetic chromosomal abnormalities in NDMM patients (8). Furthermore, there are evidence showing the effectiveness of alloSCT in overcoming translocation t(4;14) (9), del(17p13) (10), and del(13) abnormalities (11).

In addition, multivariate analysis of several research showed age as an influencing factor for survival outcome in alloSCT. In a study from the Japanese Society of Myeloma, age ≥50 years would adversely affect PFS (12). Another research showed both reduction in PFS and OS in patients >55 years (13).

All in all, there are growing evidence verifying the positive effect of alloSCT in newly diagnosed and young MM patients. Our meta-analysis aimed to provide a more comprehensive and reliable analysis to verify the efficacy and survival outcome of alloHCT in MM patients, patients in NDMM/frontline, and patients in an RRMM/salvage setting.

## Methods

### Search strategy

We conducted our search on 14/04/2023 in PubMed, Embase, Cochrane Library, and Web of Science with “(Allogenic) AND (myeloma)” as our searching term. We aimed to analyze research published in the past 10 years; thus, the publication date was limited to 14/04/2013 in all databases. All results were then downloaded to EndNote 20, and duplicated studies were removed. Studies were further filtered by their title and abstract. Full text of the remaining studies was downloaded for the final screen. In addition, studies that do not provide sufficient data were identified and removed after data extraction.

### Search criteria

The following are the inclusion criteria: (1) studies concerning patients diagnosed as multiple myeloma and have no other reported hematological disease; (2) studies that have a reported response rate and survival after allogenic transplantation (studies that did not regard allo-transplantation as their main intervention but have reported that the above data were also included in our analysis); (3) studies reported in English; (4) studies with full text.

The following are the exclusion criteria: (1) cord blood as the stem cell source; (2) insufficient reported data; (3) studies concerning pediatric cases; (4) sample size <5.

We further conducted meta-analysis in an NDMM/frontline setting and an RRMM/salvage setting independently. The study design of each study was carefully read, and studies which did not clarify research population or treatment setting were excluded.

### Statistical method

The following data were extracted: number of participants, age range, median follow-up, best response to allo-transplantation (complete remission (CR), very good partial remission (VGPR), and partial remission (PR)), and survival (1-, 2-, 3-, 5-, and 10-year OS, PFS, NRM). Our main outcome was best response rate and survival after allotransplantation in MM patients. Our secondary outcome was best response rate and survival after allotransplantation in the NDMM/frontline setting and RRMM/salvage setting.

Data were first collected in Excel, and all analyses were performed using STATA 15.1. This is a single-arm research; a random-effect model was adopted, and the results were presented in forest plot. The P value of Cochrane’s Q test <0.1 indicated the existence of heterogeneity. I^2^ statistic was used in the assessment of heterogeneity. Sensitivity analysis was then conducted. High heterogeneity (I^2^ statistic >50) was commonly observed in a single-armed study; thus, the change in I^2^ statistic after removal of a certain study was used to verify the extent of interference cause by the particular study. Sensitivity analysis could further confirm the stability of our study. Publication bias was assessed by funnel plots and was further confirmed by Begg’s and Egger’s tests. The P value in Begg’s and Egger’s tests >0.05 could confirm the absence of publication bias in our study.

## Results

**Figure 1:**
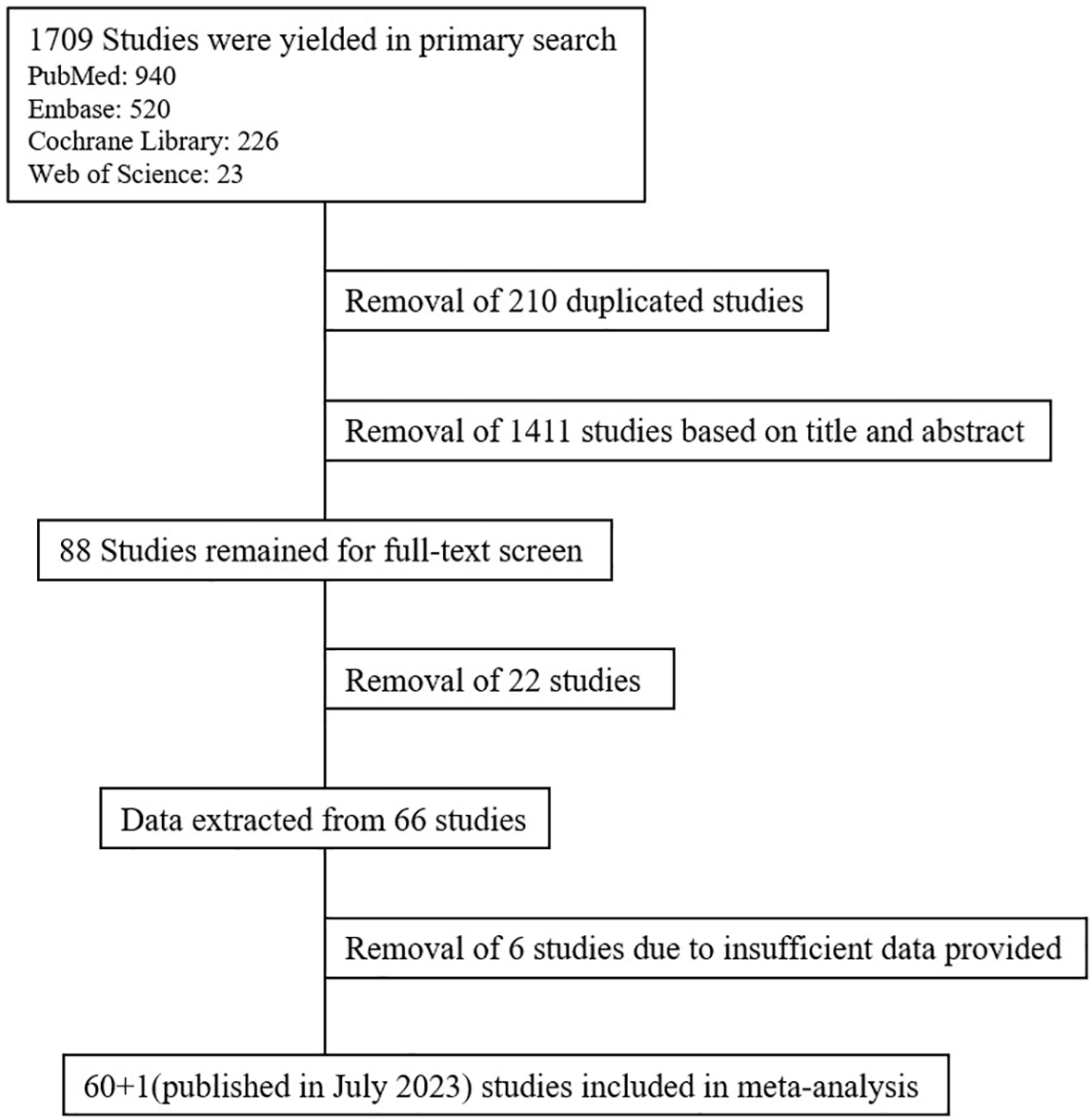
Flow diagram of the study selection.

A total of 1,709 studies were yielded from the primary search, and 210 duplicated studies were removed. Based on title and abstract, 88 studies were selected for full-text screening. After full-text screening, 66 studies remained. After data extraction, six studies were further removed as there were insufficient data provided. Additionally, we have included a study which analyzed RRMM patients who underwent alloSCT between 2013 and 2022 published in July 2023 as to enhance the veracity of the analysis. Finally, 61 studies were included in our meta-analysis (7 (11, 14–19) in 2013, 5 (5, 20–23) in 2014, 3 (24–26) in 2015, 3 (27–29) in 2016, 6 (30–35) in 2017, 4 (12, 36–38) in 2018, 7 (7, 39–44) in 2019, 12 (8, 45–55) in 2020, 7 (56–62) in 2021, 3 (63–65) in 2022, 4 (66–69) in 2023) (**Figure 1**). The overall result is summarized in **Table 1**.

**Table 1:** Summary table of meta-analysis results.

	Overall	NDMM/upfront	RRMM/salvage
Response rate			
CR	0.45 (95%Cl 0.44,0.47)	0.54 (95%CI 0.48,0.61)	0.31 (95%Cl 0.24,0.38)
VGPR	0.21 (95%Cl 0.19,0.24)	--	0.21 (95%Cl 0.14,0.29)
PR	0.24 (95%Cl 0.22,0.26)	0.22 (95%Cl 0.16,0.28)	0.12 (95%Cl 0.07,0.18)
Survival OS			
ly	0.69 (95%Cl 0.66,0.72)	--	--
2y	0.57 (95%Cl 0.54,0.59)	--	--
3y	0.45 (95%Cl 0.42,0.48)	--	0.30 (95%Cl 0.24,0.36)
5y	0.45 (95%Cl 0.43,0.47)	0.69 (95%Cl 0.65,0.73)	0.24 (95%Cl 0.21,0.28)
10y	0.36 (95%Cl 0.33,0.39)	--	0.06 (95%Cl 0.03,0.11)
PFS			
ly	0.47 (95%Cl 0.44,0.50)	--	--
2y	0.35 (95%Cl 0.32,0.38)	0.61 (95%Cl 0.49,0.73)	--
3y	0.24 (95%Cl 0.22,0.27)	--	--
5y	0.25 (95%Cl 0.23,0.27)	0.40 (95%Cl 0.35,0.44)	0.10 (95%Cl 0.07,0.13)
10y	0.28 (95%Cl 0.25,0.31)	0.32 (95%Cl 0.27,0.38)	--
NRM			
ly	0.16 (95%Cl 0.14,0.18)	--	--
2y	0.21 (95%Cl 0.19,0.23)	--	0.20 (95%Cl 0.17,0.23)
3y	0.16 (95%Cl 0.14,0.19)	--	--
5y	0.20 (95%Cl 0.18,0.21)	0.11 (95%Cl 0.08,0.15)	0.15 (95%Cl 0.11,0.20)
10y	0.15 (95%Cl 0.13,0.18)	--	--

### Response rate

**Figure 2:**
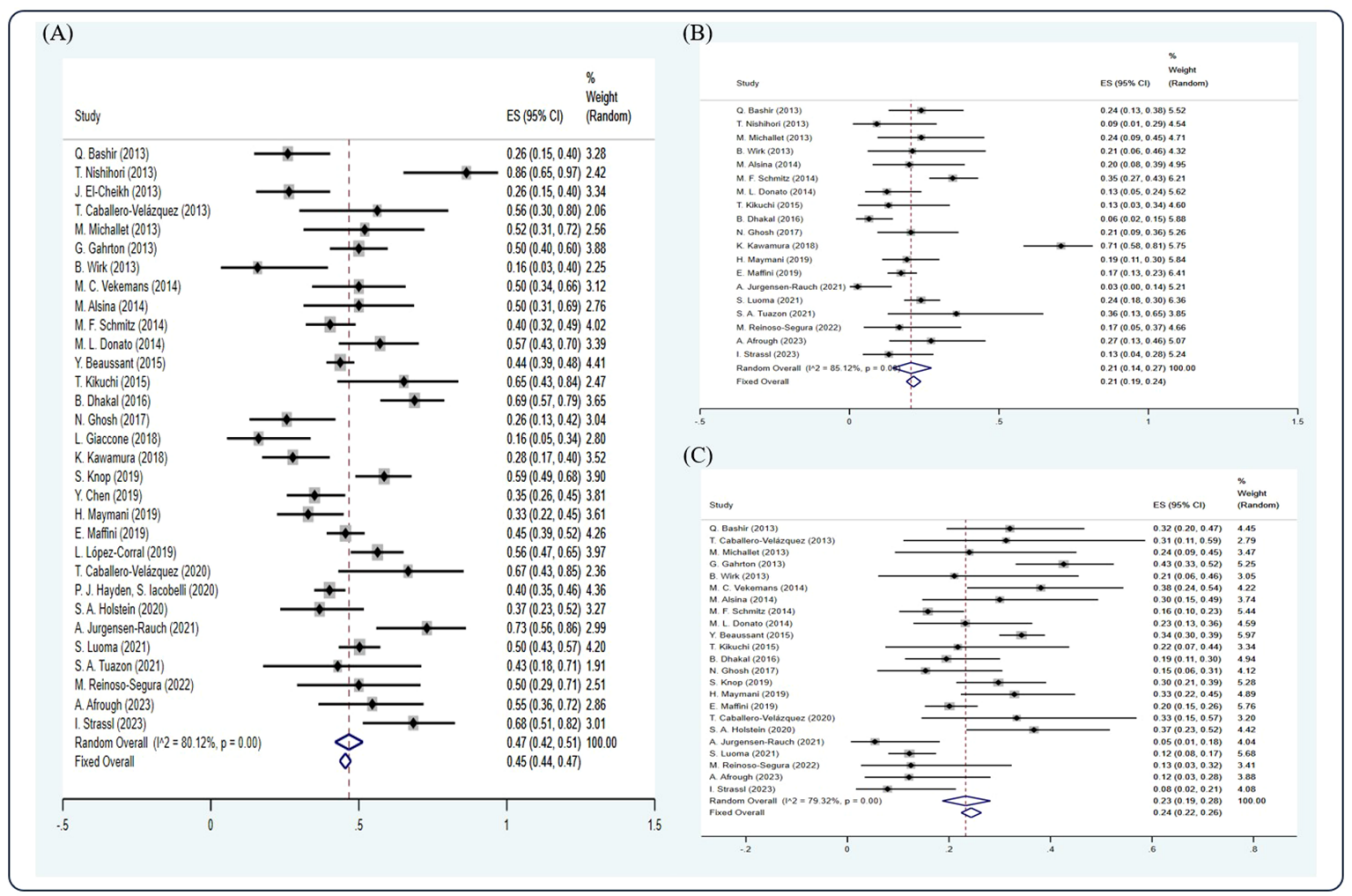
Forest plot of pooled weighted **(A)** CR, **(B)** VGPR, and **(C)** PR based on the random-effect model.

Among the studies included, 31 studies (5, 7, 11, 12, 14–21, 23, 25, 26, 29, 34, 38–41, 44, 47, 48, 54, 56, 59, 60, 64, 68, 69) have reported the CR rate, 19 studies (5, 12, 14–16, 19, 21, 23, 25, 29, 34, 39, 40, 56, 59, 60, 64, 68, 69) have reported the VGPR rate, and 23 studies (5, 7, 11, 14, 16, 18–21, 23, 25, 26, 29, 34, 39, 40, 47, 54, 59, 60, 64, 68, 69) have reported the PR rate. Based on the random-effect model, the pooled estimate CR, VGPR, and PR rates were 0.45 (95% Cl 0.44, 0.47), 0.21 (95% Cl 0.19, 0.24), and 0.24 (95% Cl 0.22, 0.26), respectively (**Figure 2**).

### Survival

#### OS

**Figure 3:**
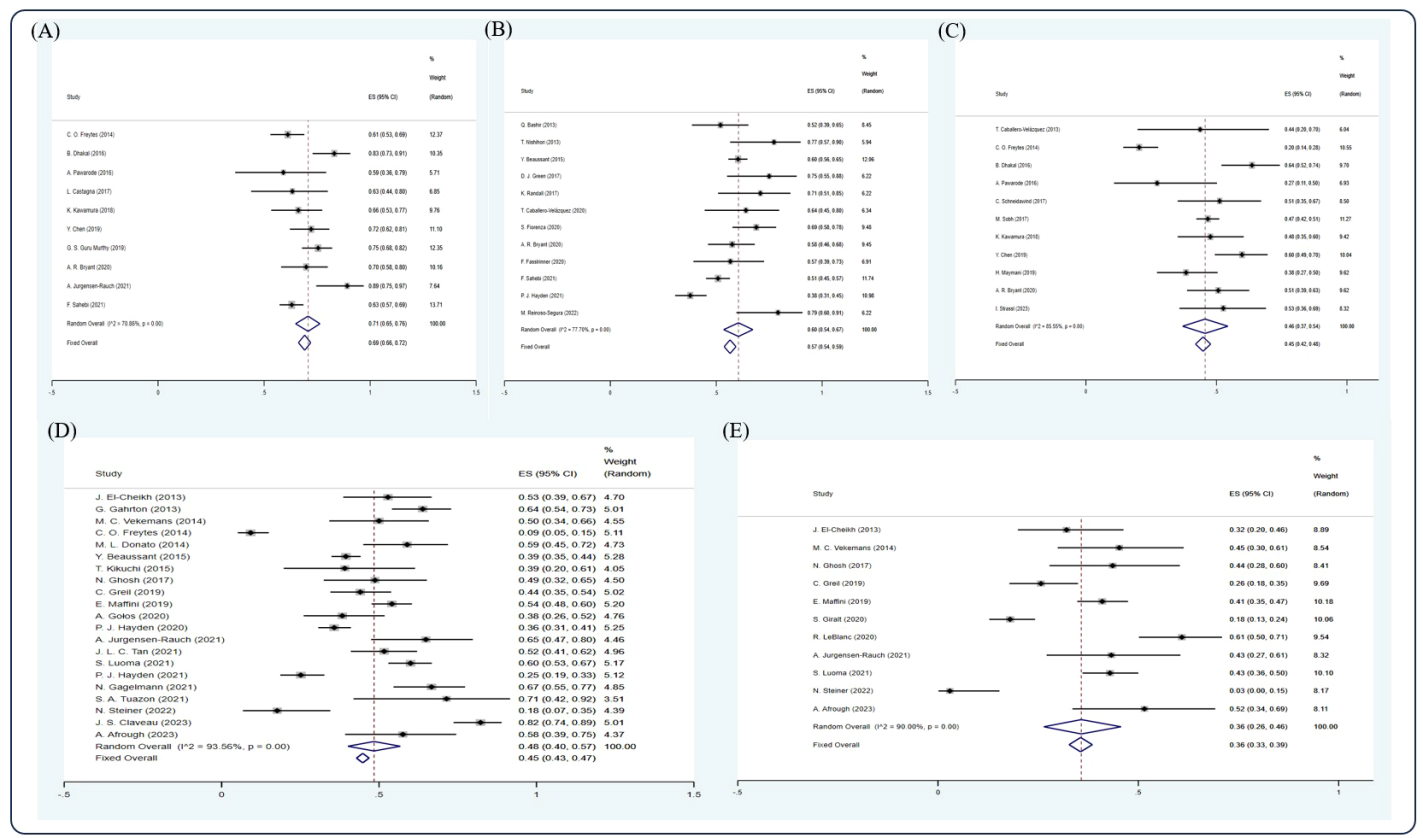
Forest plot of pooled weighted **(A)** 1-year, **(B)** 2-year, **(C)** 3-year, **(D)** 5-year, **(E)** 10-year OS based on the random-effect model.

Based on the random-effect model, the pooled estimates of 1-, 2-, 3-, 5-, and 10-year OS were 0.69 (10 studies (12, 22, 28, 29, 35, 42, 44, 55, 58, 60), 95% Cl 0.66, 0.72), 0.57 (12 studies (15, 19, 26, 32, 33, 51, 52, 54, 55, 58, 61, 64), 95% Cl 0.54, 0.59), 0.45 (11 studies (12, 18, 22, 28–31, 39, 44, 55, 69), 95% Cl 0.42, 0.48), 0.45 (21 studies (5, 11, 17, 20, 22, 25, 26, 34, 40, 43, 48, 50, 56, 57, 59–63, 66, 68), 95% Cl 0.43, 0.47), and 0.36 (11 studies (8, 17, 20, 34, 40, 43, 46, 59, 60, 63, 68), 95% Cl 0.33, 0.39), respectively (**Figure 3**).

#### PFS

**Figure 4:**
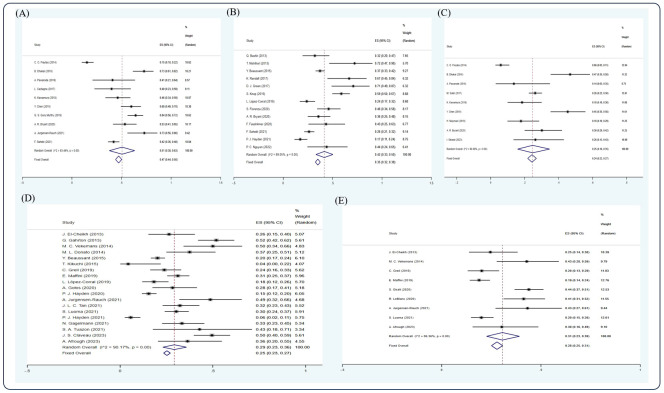
Forest plot of pooled weighted **(A)** 1-year, **(B)** 2-year, **(C)** 3-year, **(D)** 5-year, **(E)** 10-year PFS based on the random-effect model.

Based on the random-effect model, the pooled estimates of 1-, 2-, 3-, 5-, 10-year PFS were 0.47 (10 studies (12, 22, 28, 29, 35, 42, 44, 55, 58, 60), 95% Cl 0.44, 0.50), 0.35 (13 studies (7, 15, 19, 26, 32, 33, 41, 51, 52, 55, 58, 61, 65), 95% Cl 0.32, 0.38), 0.24 (9 studies (12, 22, 28–30, 39, 44, 55, 69), 95% Cl 0.22, 0.27), 0.25 (19 studies (5, 11, 17, 20, 25, 26, 40, 41, 43, 50, 54, 56, 57, 59–62, 66, 68), 95% Cl 0.23, 0.27), and 0.28 (9 studies (8, 17, 20, 40, 43, 46, 59, 60, 68), 95% Cl 0.25, 0.31), respectively (**Figure 4**).

#### NRM

**Figure 5:**
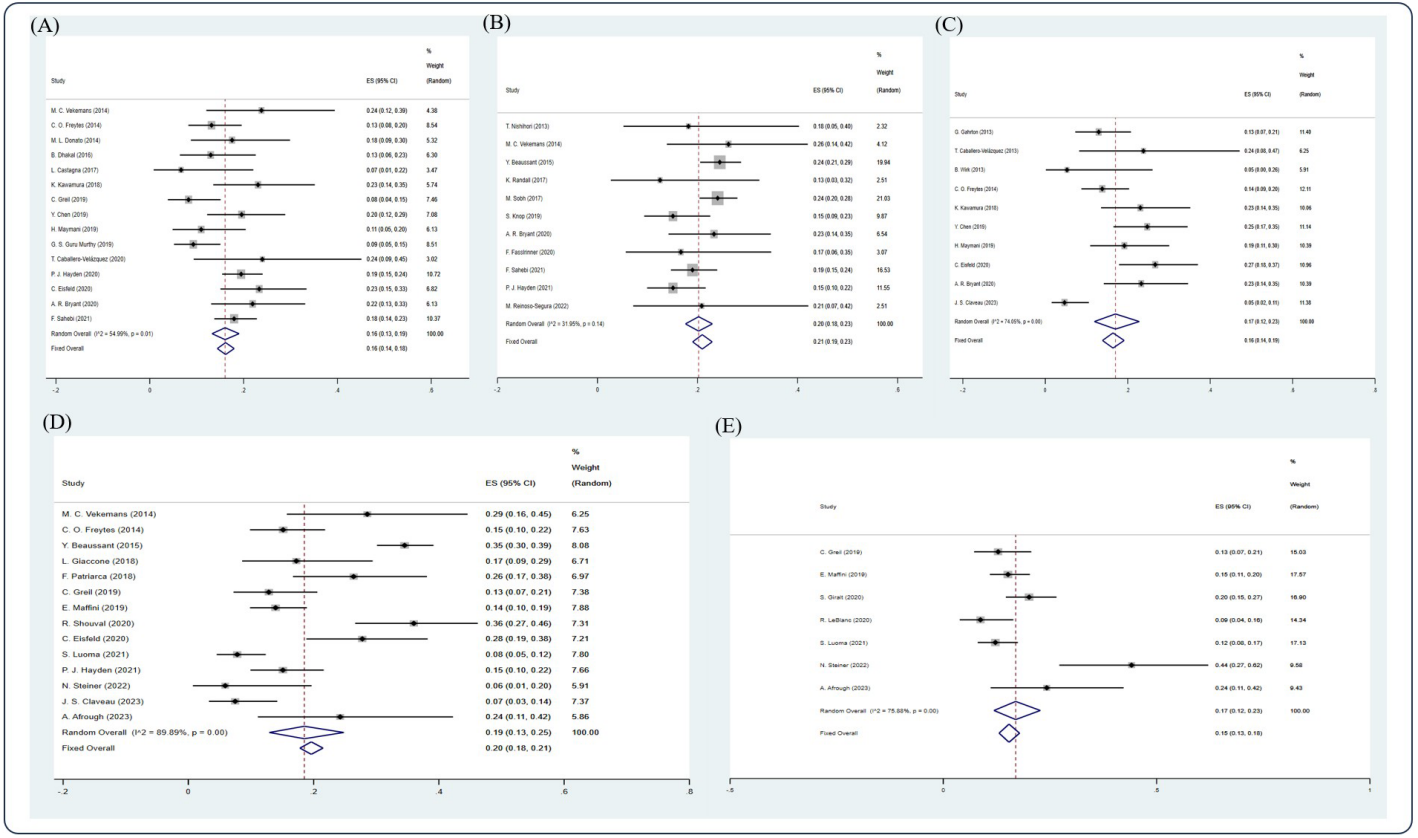
Forest plot of pooled weighted **(A)** 1-year, **(B)** 2-year, **(C)** 3-year, **(D)** 5-year, and **(E)** 10-year NRM based on the random-effect model.

Based on the random-effect model, the pooled estimates of 1-, 2-, 3-, 5-, 10-year NRM were 0.16 (15 studies (5, 12, 20, 22, 29, 35, 39, 42–44, 48, 53–55, 58), 95% Cl 0.14, 0.18), 0.21 (11 studies (7, 15, 20, 26, 30, 32, 52, 55, 58, 61, 64), 95% Cl 0.19, 0.23), 0.16 (10 studies (11, 12, 14, 18, 22, 39, 44, 53, 55, 66), 95% Cl 0.14, 0.19), 0.20 (14 studies (20, 22, 26, 36, 38, 40, 43, 45, 53, 59, 61, 63, 66, 68), 95% Cl 0.18, 0.21), and 0.15 (7 studies (8, 40, 43, 46, 59, 63, 68), 95% Cl 0.13, 0.18), respectively (**Figure 5**).

### Response rate and survival outcome in an NDMM/upfront setting

**Figure 6:**
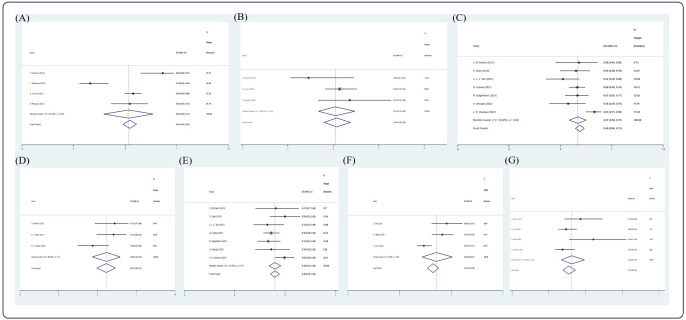
Forest plot of pooled weighted **(A)** CR, **(B)** PR, **(C)** 5-year OS, **(D)** 2-year PFS, **(E)** 5-year PFS, **(F)** 10-year PFS, **(G)** 5-year NRM in NDMM/frontline setting based on random effect model.

After screening, 13 studies (15, 17, 27, 33, 38, 43, 46, 57, 59, 62, 65, 66, 68) were included in the analysis. Due to insufficient data available, only CR, VGPR rate, 5-year OS, 2-, 5-, and 10-year PFS, and 5-year NRM could be analyzed in this section. In an NDMM/upfront setting, the pooled estimate CR rate and VGPR rate were 0.54 (4 studies (15, 38, 59, 68), 95% Cl 0.48, 0.61) and 0.22 (3 studies (15, 59, 68), 95% Cl 0.16, 0.28), respectively, whereas the pooled estimate of 5-year OS was 0.69 (7 studies (17, 43, 57, 59, 62, 66, 68), 95% Cl 0.65, 0.73). The pooled estimates of 2-, 5-, and 10-year PFS were 0.61 (3 studies (15, 33, 65), 95% Cl 0.49, 0.73), 0.40 (7 studies (17, 43, 57, 59, 62, 66, 68), 95% Cl 0.35, 0.44), and 0.32 (3 studies (43, 46, 59), 95% Cl 0.27, 0.38), respectively. The pooled estimate of 5-year NRM was 0.11 (4 studies (38, 59, 66, 68), 95% Cl 0.08,0.15). (**Figure 6**).

### Response rate and survival outcome in an RRMM/salvage setting

**Figure 7:**
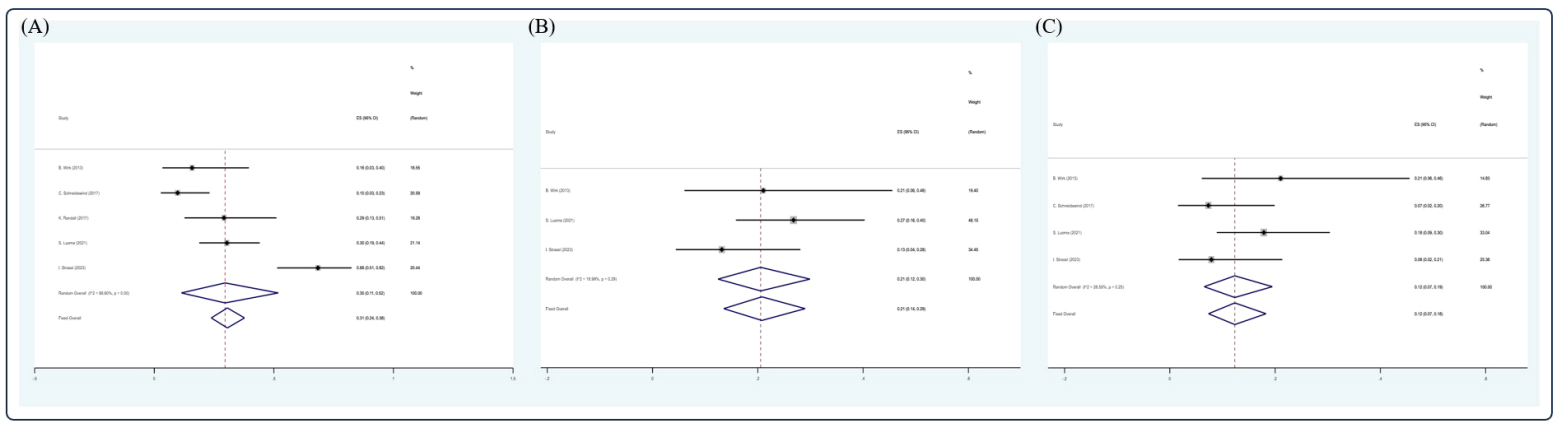
Forest plot of pooled weighted **(A)** CR, **(B)** VGPR, and **(C)** PR in RRMM/salvage setting based on the random-effect model.

**Figure 8:**
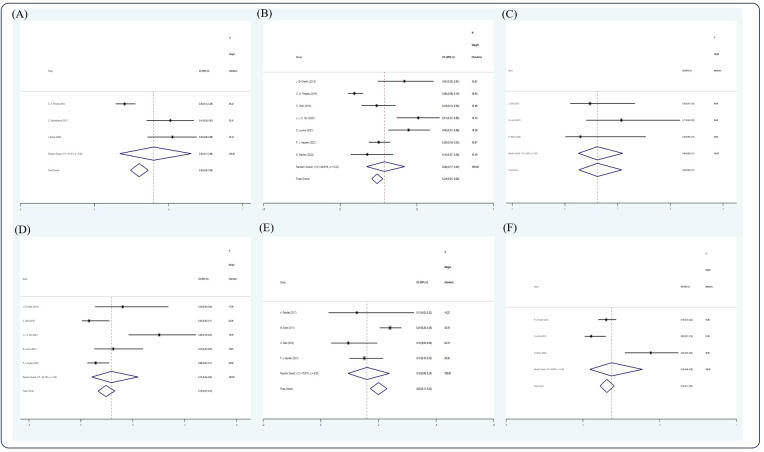
Forest plot of pooled weighted **(A)** 3-year OS, **(B)** 5-year OS, **(C)** 10-year OS, **(D)** 5-year PFS, **(E)** 2-year NRM, and **(F)** 5-year NRM in an RRMM/salvage setting based on the random-effect model.

After screening, 12 studies (14, 17, 22, 30–32, 43, 57, 59, 61, 63, 69) were included in the analysis. Due to insufficient data available, only CR, VGPR, PR rate, 5- and 10-year OS, 5-year PFS, and 2- and 5-year NRM could be analyzed in this section. In the RRMM/salvage setting, the pooled estimate CR, VGPR, and PR rates were 0.31 (5 studies (14, 31, 32, 59, 69), 95% Cl 0.24, 0.38), 0.21 (3 studies (14, 59, 69), 95% Cl 0.14, 0.29), and 0.12 (4 studies (14, 31, 59, 69), 95% Cl 0.07, 0.18), respectively, whereas the pooled estimates of 3-, 5-, and 10-year OS were 0.30 (3 studies (22, 31, 69), 95% Cl 0.24, 0.36), 0.24 (7 studies (17, 22, 43, 57, 59, 61, 63), 95% Cl 0.21, 0.28), and 0.06 (3 studies (43, 59, 63), 95 CI% 0.03, 0.11), respectively. The pooled estimate of 5-year PFS was 0.10 (5 studies (17, 22, 43, 57, 59, 61), 95% Cl 0.07, 0.13), whereas the pooled estimates of 2- and 5-year NRM were 0.20 (4 studies (30, 32, 43, 61), 95% Cl 0.17, 0.2) and 0.15 (3 studies (59, 61, 63), 95% Cl 0.11, 0.20), respectively (**Figures 7**, **8**).

### Heterogeneity analyses and sensitivity analyses

I^2^ statistic was >50% in all analyses, which indicates large heterogeneity between data sources. We failed to identify the source of heterogeneity using sensitivity analysis. Sensitivity analysis showed good stability without obvious interference by particular study.

### Publication bias

All funnel plots were visually symmetric. The P value in Begg’s and Egger’s tests of all analysis was >0.05, further confirming the symmetry of all funnel plots. No publication bias was found in our study.

## Discussion

Having a history of 66 years since the first alloSCT held by Donnall Tomas in 1957 (70), there has been an accumulation of a certain number of studies with long-term follow-up. In the past 10 years, a simple search on engines could yield 1,709 studies related to alloSCT in MM patients. The IMWG group made consensus that allogeneic SCT or tandem auto-allo-SCT should be limited to clinical trials (71), alloSCT patient data were precious and scarce. Our meta was held to optimize the use of these limited data.

In our results, OS and PFS of alloSCT, both had a marked fall from first to second and second to third years and then remained constant till the 10th year. NRM increased from the first to the second year and then remained relatively stable. The response rate of alloSCT was comparatively low. The survival benefits from alloSCT sustained. Long-term follow-up could reflect a relatively better survival outcome in alloSCT. Due to insufficient data available, the analysis of relapse rate could not be performed. The gap between OS and PFS in both settings give a clue on the survival status of patients. Furthermore, good response rate and promising survival outcome were observed in the NDMM/frontline setting.

AlloSCT could be adapted in MM patients under different conditions, as mentioned; studies suggested that it was most effective in high-risk NDMM patients. Meanwhile, there is evidence showing the beneficial effect of using alloSCT in a relapse setting, especially in young relapse patients (72). A long-term follow-up trial comparing survival outcome of relapse patients using salvage treatment with bortezomib and/or immunomodulatory agents and salvage alloSCT showed a higher 7-year OS and PFS in the alloSCT group (36). However, there is contradicting research evidence. In research aiming to compare the outcome of alloSCT in NDMM or RRMM, results showed better patient outcome in the first-line setting, with a good CR rate of 48.3%, a median PFS of 30.2 months, and a 10-year OS of 51%. The median PFS was only 8 months in the relapse setting (73). The effectiveness of alloSCT in the relapse setting is inconsistent, and age might be a more important determinant influencing the outcome.

In our meta-analysis, the CR rate and survival outcome in the RRMM/relapsed setting was below the average of the overall result, with a low CR rate of 0.31, 5-year OS of 0.24, and 10-year OS of 0.06. Due to insufficient data on survival status before alloSCT, the improvement of survival outcome could not be assessed. Despite prognostic factors such as age and cytogenetic risk, conditioning therapy and consolidation regimen could cause great influence to the result.

Since the introduction of less ablative conditioning regimens reduced-intensity conditioning (RIC) and nonmyeloablative (NMA) in 1998, NRM and systemic toxicity greatly decreased while keeping promising GvMM effects (4). Traditional myeloablative conditioning regimens such as total body irradiation, cyclophosphamide, and busulfan were less used afterwards. The working party of the EBMT had held a retrospective study comparing outcomes of MM patients undergoing alloSCT after treosulfan-conditioning (Treo), non-Treo RIC or non-Treo MAC, higher 5-year OS (Treo: 62%, RIC: 57%, MAC: 47%), and lower NRM (Treo: 10%, RIC: 17%, MAC: 19%) were observed in the group using Treo-based conditioning (49).

The use of novel agents such as PI, IMID, and ADC as post-transplant consolidation regimens have shown promising results with acceptable toxicity. The use of DLI or in combination with PI and IMID in patients relapsing after alloSCT showed sustained GvMM effects in early studies. However, GVHD was commonly observed in patients treated with DLI after SCT (74). CD19 CAR “DLI” was developed and studied in patients relapsing after allo-HCT in B-cell malignancies. Studies have shown comparatively low incidence of GVHD with promising effects (75). Besides, a case report has shown relief of myelosuppression due to rapid proliferation of BCMA CAR-T cells using auto-SCT in MM patients (76). Small sample research has shown acceptable efficacy and safety of using CAR-T in post-alloSCT RRMM patients (77, 78). In addition, Liana Nikolaenko conducted a retrospective study to evaluate GVHD of using monoclonal antibody daratumumab in a post-alloSCT setting; 41% of the 34 RRMM patients included achieved PR or better, five developed acute GVHD, and none developed chronic GVHD (79). CAR-T, antibody, or immunoconjugate may have potential synergistic immunomodulatory effect with SCT; further studies are required to verify the effectiveness and safety of treatment.

There were only a few meta-analyses with regards to alloSCT. The data were hard to organize as the fundamental state of each patient varied and there were numerous preconditioning and maintenance regimens. Our meta could only provide a comprehensive but rough overview of the relative effectiveness and trend of survival of alloSCT over the years.

## Conclusion

Our research gathered alloSCT MM data over the recent 10 years and provided a comprehensive analysis to verify the response rate and survival outcome of alloSCT in MM patients. AlloSCT has sustained OS, PFS, and NRM rates from the third year on. Long-term follow-up could reveal survival benefits of alloSCT in MM patients. Moreover, results have shown a promising effect of AlloSCT in an NDMM/upfront setting, whereas its effect in an RRMM/salvage setting is below average in our meta-analysis. Novel treatments such as CAR-T, antibody, or immunoconjugate may have potential synergistic immunomodulatory effects with SCT, whereas further studies were required to verify the effectiveness and safety of treatment.

## Data availability statement

The raw data supporting the conclusions of this article will be made available by the authors, without undue reservation.

## Author contributions

SL: Conceptualization, Data curation, Formal analysis, Investigation, Methodology, Resources, Software, Validation, Visualization, Writing – original draft. KL: Formal analysis, Methodology, Software, Validation, Writing – review & editing. XZ: Conceptualization, Data curation, Validation, Writing – review & editing. JH: Project administration, Supervision, Validation, Writing – review & editing. TL: Project administration, Supervision, Validation, Visualization, Writing – review & editing.
