# Corrigendum: Efficacy and survival outcome of allogeneic stem-cell transplantation in multiple myeloma: meta-analysis in the recent 10 years

**DOI:** 10.3389/fonc.2025.1559194

**Published:** 2025-03-21

**Authors:** Si Yu Lin, Ke Jie Lu, Xiao Na Zheng, Jian Hou, Ting Ting Liu

**Affiliations:** Department of Hematology, Renji Hospital, School of Medicine, Shanghai Jiaotong University, Shanghai, China

**Keywords:** multiple myeloma, allogeneic stem cell transplantation, response rate, survival outcome, OS, PFS

In the published article, there was an error in **Figure 5** and **Figure 6** as published. There was a tag on both figures. The corrected **Figure 5** and **Figure 6** and their captions appear below.

**Figure 5:**
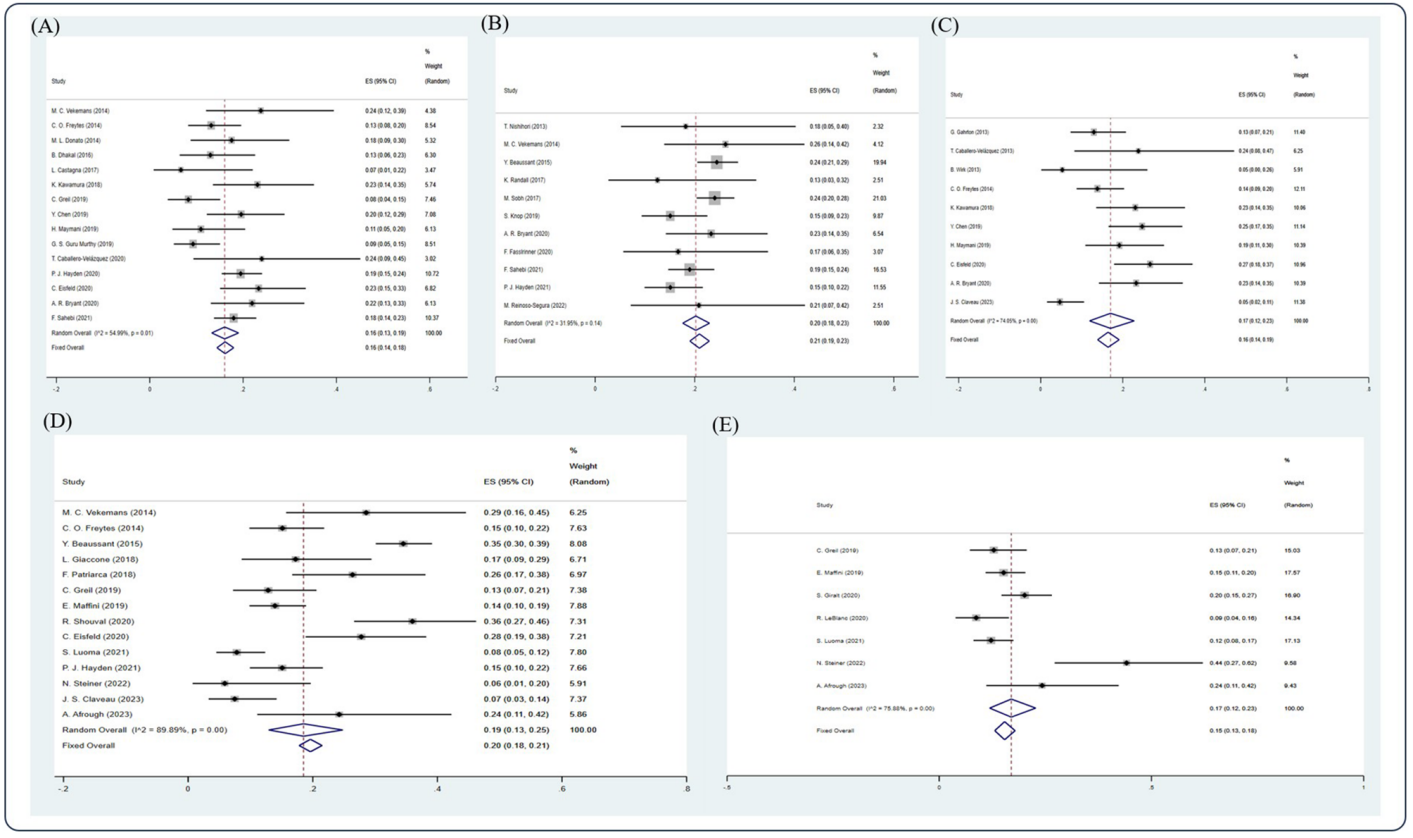
Forest plot of pooled weighted **(A)** 1-year, **(B)** 2-year, **(C)** 3-year, **(D)** 5-year, and **(E)** 10-year NRM based on the random-effect model.

**Figure 6:**
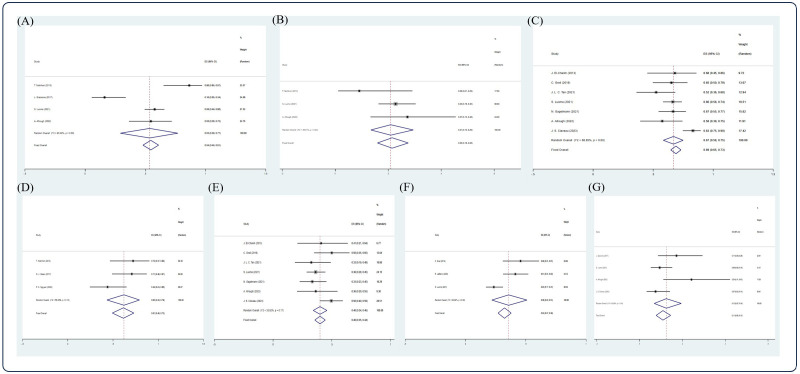
Forest plot of pooled weighted **(A)** CR, **(B)** PR, **(C)** 5-year OS, **(D)** 2-year PFS, **(E)** 5-year PFS, **(F)** 10-year PFS, **(G)** 5-year NRM in NDMM/frontline setting based on random effect model.

The authors apologize for this error and state that this does not change the scientific conclusions of the article in any way. The original article has been updated.

